# Evaluating an App-Based Intervention for Preventing Firearm Violence and Substance Use in Young Black Boys and Men: Usability Evaluation Study

**DOI:** 10.2196/60918

**Published:** 2024-11-26

**Authors:** Chuka Emezue, Dale Dan-Irabor, Andrew Froilan, Aaron Dunlap, Pablo Zamora, Sarah Negron, Janiya Simmons, Jayla Watkins, Wrenetha A Julion, Niranjan S Karnik

**Affiliations:** 1 Department of Women, Children and Family Nursing Rush University Medical Center Chicago, IL United States; 2 Humanities and Social Sciences University of Missouri–Kansas City Kansas City, MO United States; 3 Department of Psychiatry University of Illinois Chicago Chicago, IL United States

**Keywords:** telemedicine, mobile app, adolescent, violence, substance-related disorder, African American, user-computer interface, software validation, software development, mobile phone

## Abstract

**Background:**

Young Black male individuals are 24 times more likely to be impacted by firearm injuries and homicides but encounter significant barriers to care and service disengagement, even in program-rich cities across the United States, leaving them worryingly underserved. Existing community-based interventions focus on secondary and tertiary prevention after firearm violence has occurred and are typically deployed in emergency settings. To address these service and uptake issues, we developed BrotherlyACT—a nurse-led, culturally tailored, multicomponent app—to reduce the risk and effects of firearm injuries and homicides and to improve access to precrisis and mental health resources for young Black male individuals (aged 15-24 years) in low-resource and high-violence settings. Grounded in Acceptance and Commitment Therapy, the app provides life skills coaching, safety planning, artificial intelligence–powered talk therapy, and zip code–based service connections directly to young Black male individuals at risk for violence and substance use.

**Objective:**

The primary aim of this study is to evaluate the usability, engagement, and satisfaction of BrotherlyACT among target young Black male users and mobile health (mHealth) experts, using a combination of formative usability testing (UT) and heuristic evaluation (HE).

**Methods:**

Using a convergent mixed methods approach, we evaluated the BrotherlyACT app using HE by 8 mHealth specialists and conducted UT with 23 participants, comprising 15 young Black male users (aged 15-24 years), alongside 4 adult internal team testers and 4 high school students who were part of our youth advisory board. UT included the System Usability Scale and thematic analysis of think-aloud interviews and cognitive walkthroughs. HE involved mHealth experts applying the Nielsen severity rating scale (score 0-3, with 3 indicating a major issue). All testing was conducted via REDCap (Research Electronic Data Capture) and Zoom or in person.

**Results:**

Qualitative usability issues were categorized into 8 thematic groups, revealing only minor usability concerns. The app achieved an average System Usability Scale score of 79, equivalent to an A-minus grade and placing it in the 85th percentile, indicating near-excellent usability. Similarly, the HE by testers identified minor and cosmetic usability issues, with a median severity score of 1 across various heuristics (on a scale of 0-3), indicating minimal impact on user experience. Overall, minor adjustments were recommended to enhance navigation, customization, and guidance for app users, while the app’s visual and functional design was generally well received.

**Conclusions:**

BrotherlyACT was considered highly usable and acceptable. Testers in the UT stage gave the app a positive overall rating and emphasized that several key improvements were made. Findings from our UT prompted revisions to the app prototype. Moving forward, a pilot study with a pretest-posttest design will evaluate the app’s efficacy in community health and emergency care settings.

**International Registered Report Identifier (IRRID):**

RR2-10.2196/43842

## Introduction

### Background

In 2021, a total of 84.8% of children who died by firearms were male, 49.9% were Black, and 82.6% were aged 15 to 19 years, with the majority of these deaths occurring through homicide (64.3%) [1]. During the same period, Black youth aged 18 to 24 years in US cities were 20 times more likely, and Hispanic youth were 4.4 times more likely, to be fatally shot compared with their non-Hispanic White peers, highlighting a severe racial disparity in firearm-related deaths [2-8]. In 2020, homicide rates among Black male individuals aged 10 to 44 years reached levels not seen since 1994 [2,9,10], as firearm-related deaths surpassed motor vehicle accidents as the leading cause of death in children and adolescents in the United States, driven primarily by increases in firearm homicides [1]. Similarly, research consistently shows a significant association between substance use and increased risk of firearm violence. Specifically, alcohol and drug use are linked to higher odds of firearm ownership, risky behaviors with firearms, and perpetration of firearm violence [11,12].

Both firearm violence and co-occurring substance use have particularly severe consequences for young Black male individuals, who often endure the most intense psychological, physical, and psychosomatic effects related to firearm deaths and disabilities. These adverse outcomes can adversely diminish their overall well-being, health, quality of life, and future opportunities. Despite these associations, the relationship between specific substances and firearm violence is mixed [13,14].

Young Black male individuals with assault-related injuries on adverse developmental trajectories encounter unique barriers to accessing and sustaining formal support systems, including mental health, behavioral, and medical services, across a prevention, treatment, and recovery continuum [15-18]. In urban areas with many behavioral programs, minority youth exhibit a 50% to 60% drop in mental and behavioral service use after the age of 17 years [19,20]. Overcoming these barriers necessitates innovative solutions tailored to their specific daily activities, environments, and needs.

Digital health interventions (DHIs) present promising opportunities for delivering evidence-based prevention and treatment resources due to their accessibility, scalability, and potential for customization [16-19]. These interventions use digital tools and platforms to enhance health care delivery, patient outcomes, and overall well-being, which include but are not limited to smartphone apps, web-based platforms, wearable devices, virtual reality, gamification, and telemedicine systems. Similarly, several DHIs for substance use disorders have been well received by participants [21]. In contrast, current DHIs for youth and firearm violence prevention are still emergent. Existing technology-based solutions focus on secondary or tertiary prevention, such as crime control, surveillance, monitoring, offender communication, gunshot detection, and patient flagging systems [19,20]. Even so, few studies have examined the usability of DHIs for Black and African American youth [22].

Our prior research with racial minority adolescents, including gun-carrying youth, found that they prefer and are motivated to engage in technology-enhanced interventions due to their consistent delivery, confidentiality, and convenience [13,17-19,21-25]. However, these interventions are typically implemented in emergency departments (EDs) or schools, addressing issues such as bullying or cyberbullying [26], teen dating violence [27], and disruptive behaviors [28,29]. ED-based interventions such as SafERteens [21,23,30], Project Ujima [24], Helping Individuals with Firearm Injuries [25], and Healing Hurt People [31] have effectively reduced aggression, victimization, trauma recidivism, rearrests, and violence-related outcomes for up to 1 year. Despite their potential, ED-based programs encounter substantial implementation obstacles, such as staff shortages, rapid ED workflows, reimbursement constraints, emphasis on secondary and tertiary prevention, and limited geographic reach.

Social determinants of health (SDOHs), including provider biases, overcriminalization, concentrated poverty, historical mistrust of legal and medical actors, and limited educational and occupational opportunities, diminish the effects of these DHIs [15,32,33]. Structural violence, racism, and neighborhood disadvantages further exacerbate these SDOHs, discouraging young Black male individuals from engaging with prevention programs. Young Black male individuals exhibit 2 trauma-related avoidance strategies: *service avoidance*, where they steer clear of services or institutions with official records or police presence or provide false contact information to health care staff to evade criminal justice involvement [34-36], and *experiential avoidance*, characterized by suppressing unpleasant thoughts, memories, or feelings through maladaptive behaviors such as retaliation, pride in carrying weapons, firearm and other weapon-based violence, or involvement in gangs. Few interventions address these avoidance mechanisms as risk amplifiers [37-42], resulting in a revolving-door approach to firearm violence prevention, where young Black male individuals repeatedly cycle through violence-related programs without achieving lasting change. Emerging interventions must target multilevel SDOHs to create environments enabling young Black male individuals to thrive while simultaneously addressing and the psychological or cognitive risk factors that drive firearm violence [43].

### This Study

This study assesses the usability of BrotherlyACT—a nurse-led, culturally tailored, multicomponent smartphone app—to reduce the risk and effects of firearm injuries and homicides and to improve access to precrisis and mental health resources for young Black male individuals (aged 15-24 years) in low-resource and high-violence settings. Grounded in Acceptance and Commitment Therapy (ACT), BrotherlyACT addresses crisis support gaps for young Black male individuals in underserved areas. Using a stepwise methodology combining usability testing (UT) and heuristic evaluation (HE), this study identifies and refines content, functionality, and user interface and user experience (UI/UX) design to enhance usability. This user-centered, iterative approach ensures alignment with the target group’s needs and preferences, supporting the intervention’s effectiveness and user engagement.

## Methods

### Study Design

Our study used a convergent mixed methods design to evaluate the usability of BrotherlyACT. Following CONSORT-EHEALTH (Consolidated Standards of Reporting Trials of Electronic and Mobile Health Applications and Online Telehealth) guidelines, we conducted formative UT with 23 participants, comprising 15 young Black male users (aged 15-24 years), alongside 4 adult testers and 4 high school students from a youth advisory board, and HE with 8 mobile health (mHealth) and UI/UX experts from the University of Missouri’s Precision START (Smart Technologies and Applications for Rapid Translation) Lab. UT included using the System Usability Scale (SUS), think-aloud protocols, and cognitive walkthroughs, providing both quantitative and qualitative data on user experience and satisfaction. HE involved applying the Nielsen 10 Usability Heuristics [44], with experts identifying and scoring usability issues and recommending improvements for design consistency and navigation. This study and its previously published protocol [36] strictly adhered to the CONSORT-EHEALTH checklist (Multimedia Appendix 3).

### Participants: Inclusion and Exclusion Criteria

To participate in the HE, expert testers were required to have prior experience in developing DHIs and to be willing to conduct controlled intervention testing on any device of their choice. There were no predefined exclusion criteria for heuristic testers, allowing a broad range of experts to contribute their insights. Both internal and external teams conducted the UT. External testers had to meet specific criteria: they had to identify as male and Black or African American, be aged between 15 and 24 years, have internet access, and be able to provide assent or consent (including parental consent where applicable). Service providers met the following criteria: aged 18 years or older (of any gender); with prior experience working with young Black male individuals affected by firearm violence and substance use; be of any race or ethnic origin; and be able to read and write in English.

Exclusion criteria for young Black male individuals included active suicidal or homicidal behavior, being medically unstable, intoxication during recruitment, cognitive incapacity to provide informed consent, impairments (including emergent injuries), and being in police custody or incarceration during study enrollment. Internal UT testers had no specific eligibility criteria, allowing for diverse perspectives in the testing process.

### The BrotherlyACT App

#### Overview

The intervention begins with a single-session, brief motivational interviewing (MI) component (BrotherlyACT+MI) to boost young Black male individuals’ motivation, self-efficacy, retention, and treatment adherence. This brief MI-informed approach follows the Screening, Brief Intervention and Referral to Treatment model. A trained interventionist conducts the brief MI session, which is scripted for delivery by nontherapists in under 10 minutes, in a private, nonjudgmental setting. While not a full MI intervention (ie, it is MI informed), it incorporates all 4 MI principles—empathy, developing discrepancy (recognizing the gap between current behavior and future goals), rolling with resistance (anticipating pushback), and supporting self-efficacy—using 4 communication skills (ie, open-ended questions, affirmations, reflections, and summaries “OARS”). This brief session explores user goals (tracked on the app) and evokes “change talk” to personalize behavior change. Studies show MI achieves optimal results when paired with other therapies. Therefore, the MI session concludes with a warm introduction to the app, encouraging users to first complete video modules and use all app components.

BrotherlyACT integrates life skills coaching (via brief video-based psychoeducational modules), safety planning tools, artificial intelligence (AI)–powered talk therapy chatbot, and zip code–enabled service connections. This intervention is designed to support low-income young male individuals (aged 15-24 years) living in underresourced communities. It aims to reduce the risks and consequences of firearm violence and early substance use while expanding access to evidence-based therapeutic support and resources. Our app developer, Hekademeia Research Solutions, created a prototype for the BrotherlyACT app using the Quasar Framework, Vue.js, and Typescript. The app has three main components: (1) video-based psychoeducational modules, (2) a safety planning toolkit, and (3) a service engagement and talk therapy AI-powered chatbot.

#### Video-Based Psychoeducational Modules

**Figure 1:**
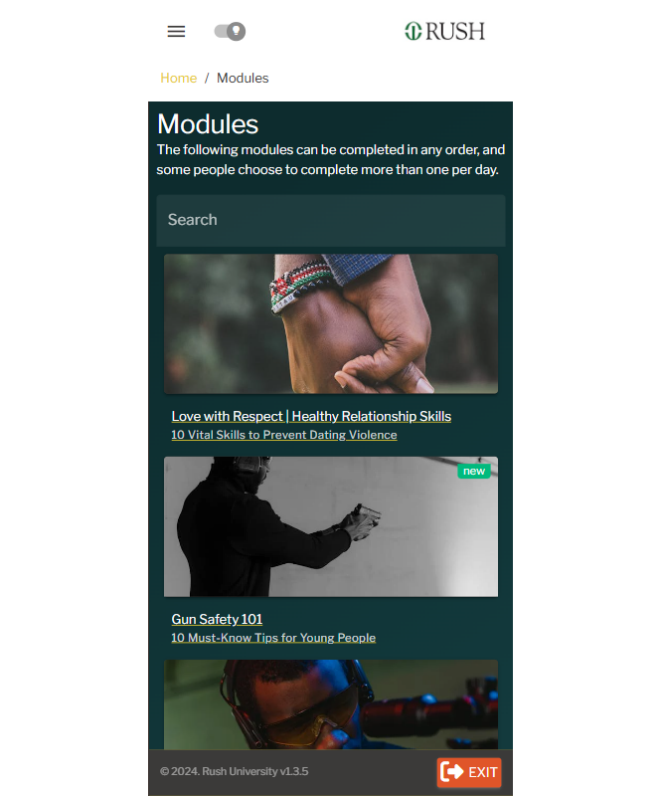
Screenshots of the BrotherlyACT prototype: psychoeducational modules.

The app provides brief psychoeducational video lessons (Figure 1) on life skills, based on the 6 core principles of ACT, a third-wave cognitive behavioral therapy. Each module takes about 5 minutes on average to complete. Modules cover skills-based topics such as gun refusal, gang resistance, gun safety, alcohol and drug refusal, conflict resolution, nonretaliation, healthy and unhealthy relationships, mindfulness (emotional reactivity regulation), fostering positive future orientation, and adaptive coping strategies. These modules feature interactive elements such as cartoons, a real-life narrator, and graphics to enhance user engagement. Multiple-choice quizzes at the end of each module reinforce and practice skills. Users can complete all lessons at once or weekly while accessing other app features, with push notifications reminding them to finish incomplete modules. Completion rewards include medallions on a pseudonymized leaderboard, with plans to connect app achievements to tangible rewards such as cash incentives.

#### Safety Planning Toolkit

**Figure 2:**
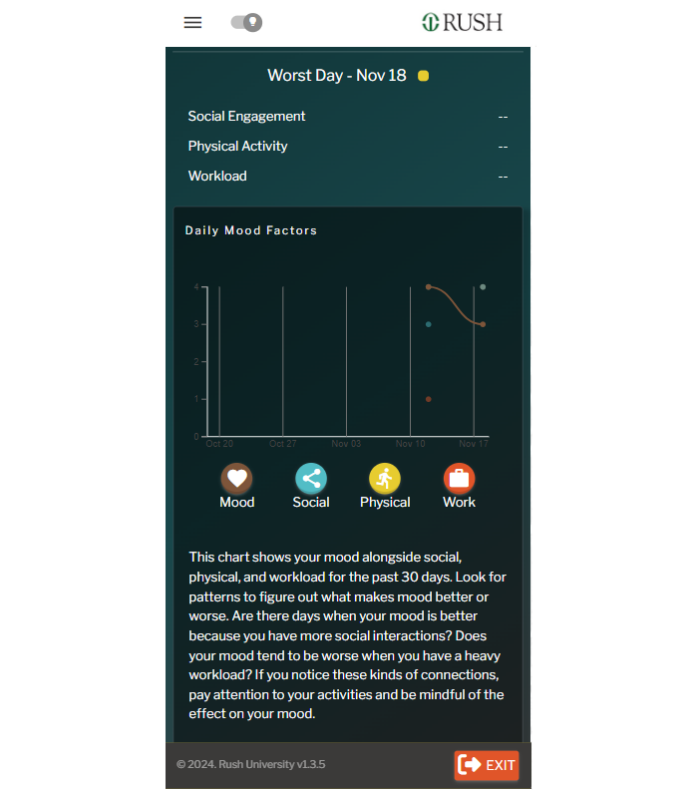
Screenshot of the BrotherlyACT prototype: mood tracker.

**Figure 3:**
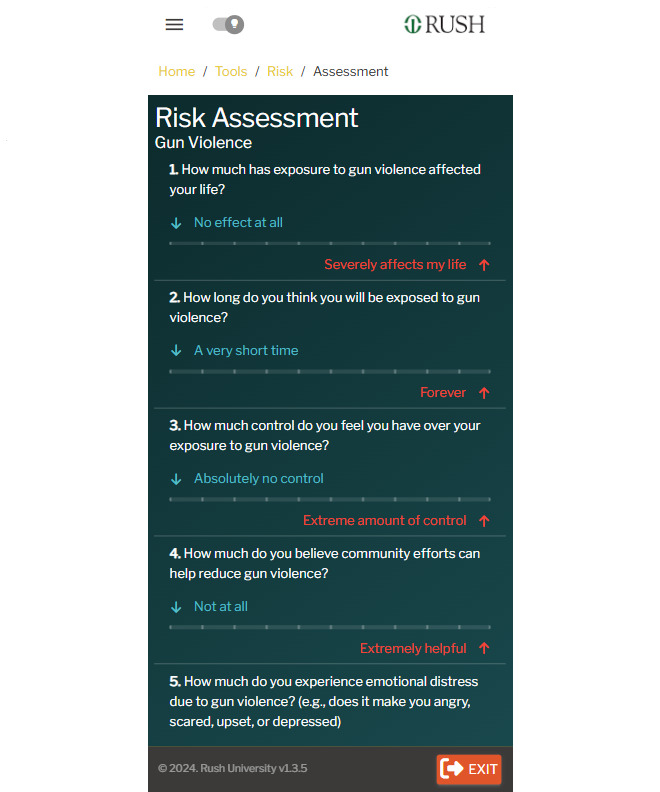
Screenshot of the BrotherlyACT prototype: risk assessment.

**Figure 4:**
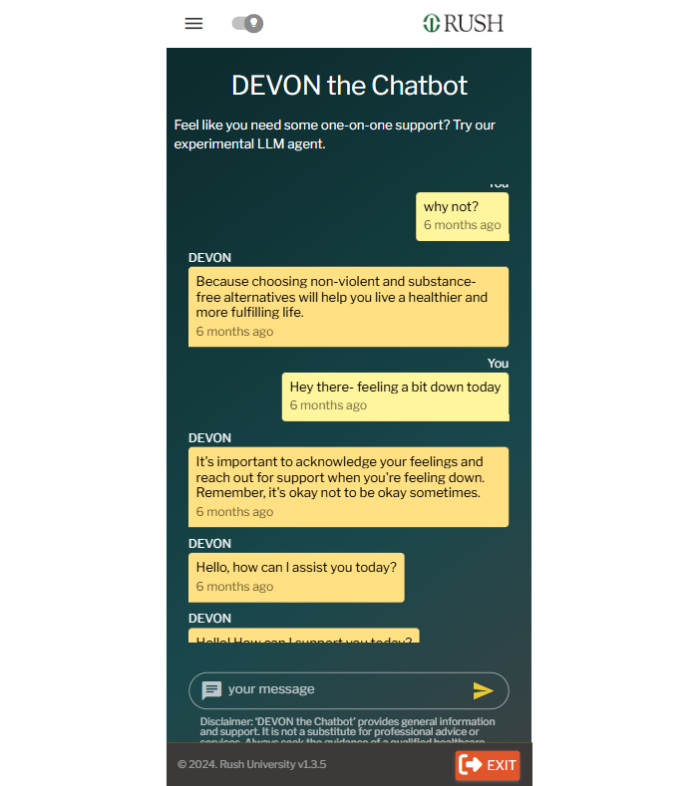
Screenshot of the BrotherlyACT prototype: Digital Empowerment and Vital Resources Outreach Network (DEVON) the chatbot. LLM: large language model.

Participants also have access to a safety planning toolkit that includes a 30-day mood trendline to track mood trends and triggers (Figure 2), and evidence-based risk assessment checklists (accessing risk for gang involvement, alcohol and drug use, and gun violence involvement using multiple choice-questions or a slide bar–weighted scoring system to create a risk score and simplified narratives explaining their risk levels; Figure 3). Participants can also access mindfulness-based stress reduction exercises (eg, guided square breathing) and a SMART (Specific, Measurable, Achievable, Relevant, Time-Bound) goal–setting feature that tracks 3 primary value-based objectives with suggested or user-defined, smaller goals. Personalized risk scores and narratives link users to preventive resources such as app modules or to the AI-powered chatbot called “DEVON (Digital Empowerment and Vital Resources Outreach Network) the Chatbot” (Figure 4). DEVON the Chatbot uses natural language processing and a question-answering system [45] powered by a deep learning model (OpenAI GPT-4.0) to provide violence and substance use reinforcement learning and talk therapy based on user inquiries.

#### Service Engagement and Talk Therapy AI-Powered Chatbot

**Figure 5:**
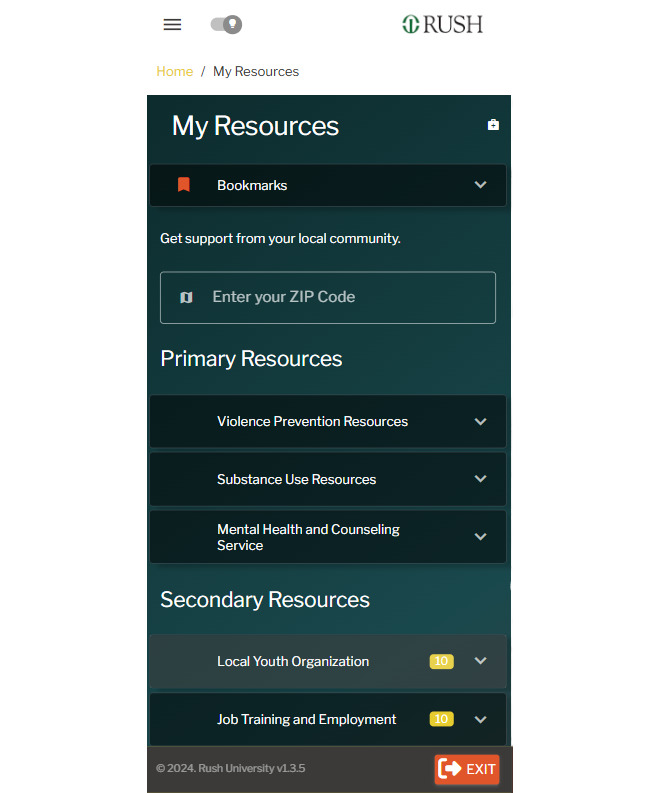
Screenshot of the BrotherlyACT prototype: zip code-based support.

The app also connects young Black male individuals to local precrisis response services and navigational support to resources within a 50-mile (80.47 km) radius, determined by the user’s zip code (Figure 5). Users are provided with a list of vetted community-level crisis services addressing practical needs (eg, jobs, housing, legal, and childcare) and mental health needs (eg, Black therapists), ensuring 24/7 support. BrotherlyACT also includes a moderator-supervised peer support chat room for young Black male individuals to anonymously connect and share advice with peers experiencing similar challenges. In our prior study, young Black male individuals requested these add-ons to foster trust in behavioral services and enhance humanlike help-seeking with either a “real person” or a relatable avatar [46].

### Ethical Considerations

Ethical and safety considerations were approved by the institutional review board of the Rush University Medical Center (Office of Research Affairs: 21122902). All participants provided informed consent or assent via REDCap (Research Electronic Data Capture; Vanderbilt University), and all data were anonymized or deidentified to safeguard participant information. Parental consent was also obtained for participants under the age of 18 years. All participants, except the 4 adult internal testers, received a US $50 e-gift card as compensation.

### Procedures

#### Methodology for UT

Between September and December 2023, UT was conducted with 23 participants, comprising 15 young Black male individuals (aged 15-24 years), 4 adult testers, and 4 high school students participating in a 3-month youth advisory board and internship program at the EMERGE Innovations Lab within the Rush University Medical Center. A dual-pronged approach was used, combining qualitative usability evaluation using think-aloud protocols and cognitive walkthroughs [47]. Both methods involved potential end users verbalizing their thoughts while actively using the app (version 1.3.1), alongside guided tasks to assess ease of use. Qualitative interviews lasting 30 to 60 minutes were conducted in person or via Zoom (Zoom Video Communications), with audio recording and screen sharing where possible. Participants expressed their thoughts on the app’s content and features using a “think-aloud” protocol, while cognitive walkthroughs used scripted tasks to identify misunderstood content or phrases. We asked broad and nontechnical questions, such as feedback on the app’s least helpful aspect and guidance on navigation. Participants accessed the app via a URL on their personal devices or study iPad (Apple, Inc) devices, differing only in screen size. Both internal and external UT teams identified which features and content to retain, modify, or eliminate. Two master’s-level research assistants conducted usability tests via Zoom using a semistructured interview guide, incorporating think-aloud techniques and cognitive walkthroughs with scripted use cases.

Next, all usability testers completed a quantitative assessment using the 10-item SUS [48,49], via REDCap [50]. The SUS is widely used to evaluate perceived usability, with studies showing a positive correlation between SUS scores and user engagement [51]. A minimum, average SUS score of 68 out of 100 is standard, with scores above 80.3 rated as excellent and in the top 10% [49,51]. The SUS is highly reliable (Cronbach α=0.90), with scores above 70 indicating usability [50,52].

#### Methodology for HE

The heuristic usability inspection occurred from April to May 2024, involving 8 mHealth expert testers experienced in developing and evaluating DHIs and related software. Each tester independently assessed the app using the Nielsen 10 Usability Heuristics, which is based on core interaction design principles [52-54]. These 8 expert testers, unfamiliar with the BrotherlyACT app, were recruited from the Precision START Lab at the University of Missouri-Columbia. They received a link to the app and were instructed to identify and assign severity ratings (using a 5-point severity ranking scale [SRS]) to areas where there were usability violations in the app. These issues were rated as follows: 0 (not a usability issue), 1 (cosmetic issue only—may be addressed if additional time is available), 2 (minor usability issue—priority should be given to resolving it), 3 (major usability issue—should be given high priority for resolution), and 4 (usability catastrophe—requires immediate action to rectify before product release). The testers could explore all app sections freely, without predefined use cases, unlike during the UT phase. In addition, heuristic testers provided written, open-ended narratives for each heuristic on REDCap to explain the issues they identified and offered recommended solutions. Examples of HE questions include “What were the issues you identified with the ‘Visibility of System Status’, and what are your recommendations?”

### Data Analysis

#### UT Analysis

Participants answered 5 pairs of contrasting questions in the 10-item SUS, each with one positive and one negative wording. For the SUS score, sums of odd-numbered (negative) questions formed *X*, and sums of even-numbered (positive) questions formed *Y*. The equations used to calculate the SUS was *X_0_ = X – 5* and *Y_0_ = 25 – Y*. We calculated the SUS score by multiplying *X_0_* and *Y_0_* by 2.5. To convert the original scores of 0 to 4 to a 0 to 100 scale, we assigned each question a point value (eg, strongly disagree was given 1 point), added the points together, and multiplied by 2.5. Although scores can range from 0 to 100, they are typically ranked on a percentile basis. An SUS score >68 is considered above average [48]. We conducted a thematic analysis of qualitative UT data using a study-specific codebook divided into 8 categories: app navigation, module content, user interface design, app functionality, app interactivity, app user experience, cultural referencing, and miscellaneous codes. The lead author and 2 research assistants (AF and AD) performed inductive coding on the transcripts, applying this predetermined coding structure to evaluate necessary app modifications.

#### HE Analysis

After UT, mHealth specialists conducted an HE of BrotherlyACT using the Nielsen 10 Usability Heuristics, rated on a 5-point SRS. The 10 heuristic categories were divided into 22 subcategories, with expert recommendations and design solutions suggested for each. Evaluators examined system behaviors that violated the subheuristics. The Nielsen 10 Usability Heuristics form was adapted from a publicly available HE Workbook by the Nielsen Norman Group. Our objective was to identify and address any severe issues with scores of at least one on the SRS.

## Results

### Participant Characteristics

A total of 23 participants, comprising 15 young Black male individuals (aged 15-24 years), 4 high school students, and 4 adults, participated in our formative UT. The average age of the participants in the young Black male group was 21 (SD 5.74; range 15-24) years. The mean age of the participants in the high school group was 17 years, and all 4 adult testers were older than 30 years. The 15 young Black male individuals in our sample were selected from a larger randomized controlled trial registered at ClinicalTrials.gov (NCT06359990), where the average age was 18.21 (SD 3.25) years, with most participants (68/312, 23.7%) being aged 15 years. All participants in our study were Black or African American, and only a small percentage identified as multiracial or Hispanic or Latinx (27/312, 11.34%). Most participants reported having some high school education (133/312, 42.7%), having a high school diploma (82/312, 26.3%), or being currently enrolled as students (179/312, 57.3%). In addition, 19.6% (61/312) of the participants worked part-time, which amounts to ≤35 hours per week.

DHI experts (n=8) at the University of Missouri-Columbia’s Precision START Lab conducted our HE. The sociodemographic profile of DHI expert testers included 50% (4/8) identifying as White and 25% (2/8) identifying as Asian. Most had a PhD or professional degree (5/8, 60%), were female (4/8, 56%), and identified as assistant professor (4/8, 55%). The remaining expert testers comprised individuals with various functions: a data analyst and a PhD candidate in health informatics; a postdoctoral fellow; a nurse practitioner specializing in primary care, addiction, and chronic pain; a PhD student in nursing science; and a registered nurse.

### UT Quantitative and Qualitative Feedback

The BrotherlyACT app achieved an average SUS score of 79, indicating near-excellent usability. This score corresponds to an A-minus grade and ranks the app in the 85th percentile for usability. All usability testers held a positive view of the prototype, giving it a score of 8 out of 10 (0=not at all likely and 10=extremely likely) for their level of satisfaction and the likelihood of recommending it to others. The qualitative interviews uncovered issues with the app that needed to be addressed, leading to 46 pages of codes and subcodes organized into 8 thematic areas: app navigation, module content, user interface design, app functionality, app interactivity, app user experience, cultural referencing, and miscellaneous codes. Participant feedback within these 8 thematic areas was categorized as negative, neutral, or positive (praising sections of the app), with some codes falling into more than one thematic area. The highest frequency of feedback was found in the “app user experience” area, which included a mix of positive and critical comments. Some substantial usability issues were that participants preferred real-life videos over the original cartoon videos; wanted characters in the videos to reflect them; and noted complications with using the app, with several features not particularly arranged to improve navigation. In response, we addressed the numerous usability issues they brought up. Importantly, all prior videos were revamped to feature a Black narrator and images that better reflected Black culture and the lived experiences of young Black male individuals. Users also requested a brief video explaining how to use the app, in place of the prior text description. In addition, daily reminders were recommended to ensure progress through the app sections. Adolescents found *My Modules* (ie, video-based psychoeducational modules) and the mindfulness-based stress reduction tools to be the most useful features of the app. Minor issues with other components of the app are organized into 8 thematic areas (Multimedia Appendix 1). A previous study captured predesign needs from young Black male individuals as target users between July 2022 and 2023 that went into this version of the app, and another study evaluated intervention “must-haves” for young Black male individuals impacted by bot violence and substance use [46]. We summarize individual feedback from our UT participants.

### HE Feedback

Eight mHealth and UI/UX experts performed an HE on the app. Most of the app’s usability heuristics received a median score of less than 1, indicating no problems existed, showing the severity ranking results from our expert testers (Multimedia Appendix 2). The “Help users recognize, diagnose, and recover from errors” and “User control and freedom” heuristics had no violations. However, the “Help and documentation” heuristics had the highest violations, with a median score of 1.5. Previous research suggests that mHealth experts, due to their familiarity with digital platforms, tend to assign higher severity scores compared with unfamiliar testing groups, as was the case with our UI/UX experts [47]. Considering the feedback received, further actions were taken to address the concerns raised during the HE.

Specific issues identified were primarily navigation and customization issues within the prototype, such as missing back buttons, missing undo and redo buttons, iconography choice, the need to make pages better navigable, the need for menu item relabeling (eg, relabeling “My Modules” to “My Lessons”), and the addition of a subpages list to the main menu list. HE evaluators were also concerned about the “Exit” button, relabeled as “Quick Exit,” and lauded this feature as a crucial safety and security element. Evaluators praised the style and content but struggled to navigate the “My Dashboard” panels (containing features such as My Mood Tracker, a quick overview of modules, goal trackers, ranking on a leaderboard, and a panel with daily positive quotes). Similarly, issues of output comprehension and navigability were noted with the *Mood Tracker*. We used the findings from the HE to refine the prototype and make it more user friendly.

## Discussion

### Principal Findings

In this convergent mixed methods study, we assessed the usability of BrotherlyACT, a novel nurse-led, culturally tailored, digital intervention aimed at reducing youth and firearm violence and co-occurring substance use risk among young Black male individuals (aged 15 to 24 years). Through a stepwise methodology integrating HE and formative UT, we identified and refined content, functionality, and UI/UX design elements to optimize usability. BrotherlyACT achieved an average SUS score of 79, equivalent to an A-minus grade and placing it in the 85th percentile, indicating near-excellent usability. HE further validated these findings, with mHealth expert testers reporting minor errors and a highest severity score of 1.5 on a scale of 0 to 3 (with 3 indicating a major issue), underscoring that the app is well designed, user-friendly, and ready for effective use by its target audience with minimal adjustments needed. HE results showed that most usability heuristics received median scores of less than 1, indicating no key issues. However, the “Help and documentation” heuristic had the highest violation score of 1.5. Feedback highlighted navigation and customization challenges, such as missing buttons and unclear labeling.

Qualitative UT interviews revealed some usability issues categorized into 8 thematic areas, including app navigation, user interface design, and cultural referencing. Key concerns included a preference for real-life videos featuring relatable characters, navigation challenges, and the need for instructional content on app use. In response, we revamped the app’s video content to feature Black narrators and added a user-guided how-to video. We have used this feedback from the UT and HE stages to refine the app prototype, improving its usability and ensuring it better meets the needs of young Black male individuals at risk for violence and substance use. To our knowledge, BrotherlyACT is the first evidence-based app addressing youth firearm violence and substance use using an ACT-based approach. These usability findings highlight its potential as a practical, effective clinical tool for engaging a high-risk, underserved population.

Recent studies have explored UT for violence prevention DHIs, particularly in the context of intimate partner violence and sexual assault. mHealth apps have shown promise in addressing similar interpersonal violence issues, with usability evaluations indicating high user acceptance and effectiveness in screening, disclosure, and prevention [55-57]. Various usability evaluation methods, including questionnaires, interviews, and think-aloud protocols, have been used to assess these types of interventions [56]. However, existing DHIs for youth violence prevention have typically focused on survivors of partner or dating abuse [58,59], suicide prevention [60,61], surveillance and monitoring of youth cohorts [62], and technology-enabled violence against women and girls [62]. Earlier computer-based programs, such as SMART Talk, were successful in reducing beliefs supportive of violence and increasing intentions to use nonviolent strategies among middle school students [63-65]—often addressing internalizing and externalizing risk factors [56]. Other studies using multimedia to teach life skills (such as the Botvin Life Skills Training [66,67]) have shown that a comprehensive approach, including material on violence and the media, anger management, substance use, and conflict resolution skills, can be effective in reducing violence, delinquency, and drug use among students. These findings further highlight the potential of DHIs in primarily preventing youth violence and its antecedents [66,67], as well as in preventing substance use initiation and associated consequences [68-71].

Building on this precedence, implementing BrotherlyACT can effectively reduce barriers to care that have historically impeded service use among young Black male individuals by providing continuous access to evidence-based resources and leveraging web-based or digital tools to close key equity gaps—an area of distinct need in the violence prevention literature [58]. Given the prevalence of structural racism and racial trauma impacting these youth, BrotherlyACT addresses critical gaps in mental health support, positioning itself as a primary prevention tool. By intervening before violence occurs, the app focuses on pivotal moments such as the onset of firearm carriage or early substance use—key turning points that can escalate from low-level aggression (eg, physical fighting and bullying) and psychological distress to more severe, high-lethality violence and substance use disorders [59]. The positive results from this UT and HE study set the stage for a pilot study to assess BrotherlyACT’s clinical efficacy in community health and emergency care settings. Specifically, by addressing firearm injuries and fatality risk outcomes such as attitudes toward guns and violence, reactive and proactive aggression, mental health outcomes (eg, psychological distress), and substance use (ie, onset, frequency, and attitudes).

This study, although insightful, has limitations. The sample size, while sufficient for UT, may not represent the diversity of user experiences within at-risk young Black male populations. Our UT and HE were conducted in controlled environments, which may not reflect real-world conditions, such as in crisis situations. Future research should include more diverse samples and consider longitudinal assessments to evaluate long-term usability in real-world settings. User engagement and app functionality may change over time, revealing issues not evident in early testing stages. Factors such as internet connectivity, device types, app compatibility, data privacy, and users’ physical and emotional states could affect app usability and engagement—these dimensions may warrant further field testing in natural settings to provide ecologically valid insights into the app’s performance vis-à-vis user experience.

In addition, this phase of our study did not explore the app’s efficacy in changing behavioral, cognitive, and mental health outcomes or its integration with existing support systems (ie, health care and community behavioral health settings). A pilot study is currently under way to fully understand the app’s clinical utility and efficacy in community health and emergency care environments. While BrotherlyACT shows promise as a digital intervention for at-risk young Black male individuals, addressing these limitations will be essential for optimizing its effectiveness and ensuring broader applicability and sustainability. The app’s cultural tailoring is a strength but may limit its applicability to other groups impacted by firearm violence (eg, American Indian and Alaska Native youth). The study’s main strength includes our use of a rigorous, convergent mixed methods approach and a comprehensive, multiphase UT protocol with multiple end-user groups with varying familiarity with DHIs.

### Conclusion

In conclusion, BrotherlyACT shows promise as a user-friendly, culturally relevant, digital intervention to reduce youth violence and substance use among young Black male individuals. Positive usability scores and feedback from users and experts highlight the app’s potential to fill a critical gap in preventive care. With minor revisions and further testing, BrotherlyACT could be a vital tool in mitigating violence and promoting health equity within this vulnerable population. Adolescents and experts reported the benefits of this type of intervention for violence-involved young Black male individuals in clinical and community health settings. By enabling young Black male individuals to practice essential life skills (eg, emotional regulation, anger management, conflict resolution, and perspective taking); create a safety plan; and connect with reliable services, these types of interventions can empower young Black male individuals to manage risky behaviors and access tools and resources despite prevailing risk factors. Clinicians find the app useful for reaching susceptible youth from a primary prevention perspective and recommend using it alongside existing services as a take-home intervention. They note its benefits for both “treatment veterans” and those who are “treatment curious” due to its focus on ready-to-use life skills and behavior activation. Further investigation is necessary to assess BrotherlyACT’s effectiveness in addressing the outcomes outlined in our previously published protocol [36], primarily through implementation studies with larger youth samples in clinical and community-based settings.

## Appendix

Multimedia Appendix 1 Summary of feedback generated from young Black male individuals during usability testing.

Multimedia Appendix 2 Severity ranking scale results from expert testers.

Multimedia Appendix 3 CONSORT-EHEALTH checklist (V.1.6.1).

## Abbreviations

ACT: Acceptance and Commitment Therapy
AI: artificial intelligence
CONSORT-EHEALTH: Consolidated Standards of Reporting Trials of Electronic and Mobile Health Applications and Online Telehealth
DEVON: Digital Empowerment and Vital Resources Outreach Network
DHI: digital health intervention
ED: emergency department
HE: heuristic evaluation
mHealth: mobile health
MI: motivational interviewing
REDCap: Research Electronic Data Capture
SDOH: social determinant of health
SMART: Specific, Measurable, Achievable, Relevant, Time-Bound
SRS: severity ranking scale
START: Smart Technologies and Applications for Rapid Translation
SUS: System Usability Scale
UI/UX: user interface and user experience
UT: usability testing

## Data Availability

The data supporting the findings of this study are available from the corresponding author upon reasonable request. Due to the small sample size and identifiable affiliations with specific institutions, data access is restricted to protect participant confidentiality. Requests for data access will be reviewed in accordance with institutional policies on data sharing and participant privacy.
