# Supplementary material for: Evaluating an App-Based Intervention for Preventing Firearm Violence and Substance Use in Young Black Boys and Men: Usability Evaluation Study
Source: JMIR Form Res. 2024 Nov 26;8:e60918. doi: 10.2196/60918 (PMC11632291; doi:10.2196/60918)
Supplement: Multimedia Appendix 1 [file formative_v8i1e60918_app1.docx]

| Usability issues identified | Thematic definition of usability issues | Frequency of codes^a^ | Exemplar quotes from participants |
| --- | --- | --- | --- |
| App navigation | This code assesses the usability of navigating multiple sections or pages of an interface, including evaluations of main menus, submenus, back and forward buttons, and the functionality of links and buttons within the interface. | 42 | - “It’s very easy to navigate. Very easy to access.” [P55] - “Yeah. it’s easy to use though. It’s simple kind of. But I think being redirected out of it could be something I wouldn’t want to see in the future.” [P57] - “Well that’s just a bit I can remember, but this mood tracker, I was just navigating through it and I didn’t really understand it, but if I have more time, there would be more I would do this.” [P63] |
| App modules content | This code pertains to any proposed modifications for improving the learning modules or lessons. | 108 | - “A video, a real image will be better for the video. I think using a real image will be very preferable than an anime. So I think using real images would be very preferable.” [P55] - “I’m not really someone that does meditation. But the fact that it doesn’t just introduce you to meditation, it doesn’t just tell you. It actually tries to teach you about why you need it or define it and what it does, what it hopes to achieve, and, you know, all those mindfulness things. What I also noticed is I kept getting out of the app into like… because it takes me to some websites.” [P57] - “The tutorial video at the beginning, because there was a place that they asked me to even I wanted to learn how to use the app, so I tapped, I think it was yes. And then the video popup disappeared. I don’t know where it went, but I didn’t watch any tutorial video that I was hoping to watch.” [P60] - “It is actually a great idea because a lot of Black people out there are facing a lot of issues, a lot of young Black men domestic issues concerning legal rights abuses, substances, substance abuse facing a lot of mental health issues, out there facing discrimination. And they’re trying to like quit. Like they’re trying to quit using a lot of substances and a lot of them are addicted to substances and like a lot of them are also like, facing domestic issues. So I think an app specifically designed to meet these needs for younger people is actually something that it will be very, very useful.” [P60] |
| App user interface design | This code focuses on the overall design elements of the interface. It includes subcodes such as layout, typography, color scheme, and consistency of design elements. Evaluating this code involves assessing the visual appeal and usability of the interface. | 21 | - “Yeah. It’s easy to use though. It’s simple kind of. Okay. But I think, I think being redirected out of it could be something I wouldn’t want to see in the future.” [P57] - “More appropriate for someone my age. Just make it with a more simple user interface, more just simple not too much features, like, not, not like features, like not too much unnecessary features in it, but just simple, plain and beautiful user interface that, that would actually be more appropriate for someone my age.” [P60] - “So, and this was quite complicated to use, but my activities and tools, I like the part where I got to see good everyday course of the days and, but then what I like about just one is if this update pops up like every morning at say 7:00 AM or at specific times. So that was one thing I wanted to add in this part.” [P63] |
| App functionality | This code assesses the technical performance of the interface, which incorporates subcodes such as responsiveness, error handling, loading times, and accessibility features. | 12 | - “Nah, I saw badges like the more you do a certain thing, the more like they, they reward you the badge. That was actually great. Nice idea and good suggestions. But the application had some few glitches, some few technical error that I encountered. Like when I tried to open the application, there was a popup asking me my date of birth, my ethnicity and my gender. So whenever I wanted to type in my gender or my ethnicity, every other thing else would be like refreshed and just continued in that loop. Like I, I wasn’t really able to type, so I just keep it like it was actually on the popup of the face page when you working the app.” [P62] - “And then there could also be a notification that reminds you that, okay, you haven’t done this. So for instance, today’s date is 23rd. Yeah. So if by 23rd I didn’t complete the [module], I get a notification telling me, or a reminder say every hour that I haven’t opened a page of the [module]. And then every time I’m completing that goal, I can input my progress and say, okay I’ve done one or two and that was that.” [P63] |
| App interactivity | This code assesses user interaction with the interface, including feedback mechanisms, engagement features, and effectiveness of interactive elements such as forms or quizzes. | 22 | - “There’s something else I wanna add about the mood thing. It is not, it’s not enough just to get my mood.” [P57] - “When you just getting the app send you a notification and say, Hey, even if it’s just giving you a peaceful reminder, you know, them little, them little affirmations help sometimes just pop up during the day just to give you a reminder. Absolutely. what your goal is of the day. So yeah, I would most definitely have that as part of the app where whatever it is, they could schedule whatever it is for the app to send them notifications for different stuff during the day. Whether it, hey, if they say I might schedule to meditate two times a day and I can have the app to not notify me. Meditation, take 15 minutes out, meditate. You know, so yeah. I like that future.” [P65] |
| App user experience | This code encompasses the overall impression and satisfaction of users with the interface. It includes subcodes such as overall impressions, ease of use, user satisfaction levels, and specific suggestions provided by users for improving their experience with the interface. | 228 | - “More ideas. Yeah, more resources and more ideas should be added. Like more values.” [P55] - “I’m very much interested in referring this app to people, and I think you guys need to do a population and to, to get this app to every youth. Especially in our community and in our state.” [P55] - “I think testimonies should be good because testimonies from previous users who actually talk about how helpful it was when they used this. Kind of like a review. A place for review. Kind of things like that. It could give someone who wants to look at it extra confidence to try to use it to help his whole self.” [P57] |
| Cultural referencing | Direct or indirect reference to specific elements of the app reflecting Black culture (race, socioeconomic status, language, other culture-based experiences, etc) that may impact their engagement. | 104 | - “Okay. I wouldn’t want to choose listening because I don’t know the pace. Yeah. And I don’t know, I don’t know if I’m gonna understand, you know, we, we have, we have some cases where I don’t understand from listening because maybe the, the way the person speaks or if the person has some accents, I already understand. So for, for that reason alone, I would go for reading.” [P57] - “It is actually a great idea because a lot of Black people out there are facing a lot of issues, a lot of young Black men domestic issues concerning legal rights abuses, substances, substance abuse facing a lot of mental health issues, out there facing discrimination. And they’re trying to like quit. Like they’re trying to quit using a lot of substances and a lot of them are addicted to substances and like a lot of them are also like, facing domestic issues. So I think an app specifically designed to meet these needs for younger people is actually something that it will be very, very useful.” [P60] - “So if I actually wanna do this to be more inclusive to everyone and then my beliefs as a Christian I think that if it offered resources at Christian youth or Christian young black males actually would be a great idea because you will want to reach out to as many people as possible... And also images, like going through it especially in the, in the skill section, going through it and you see some background images being people of different demographics, people of different faiths, beliefs, practices, it shows that pretty much everyone is covered. Everyone is welcomed.” [P60] |
| Miscellaneous codes | Emerging codes that are not on the list mentioned earlier. | 4 | - “Okay. I wouldn’t want to choose listening because I don’t know the pace. I don’t know if I’m gonna understand, you know, we have some cases where I don’t understand from listening because maybe the way the person speaks or if the person have some accents, I already understand. Mm. So, for that reason alone, I, I would go for reading.” [P57] |

^a^The number of times participant’s feedback fell under each thematic area.
