# Supplementary material for: Evaluating an App-Based Intervention for Preventing Firearm Violence and Substance Use in Young Black Boys and Men: Usability Evaluation Study
Source: JMIR Form Res. 2024 Nov 26;8:e60918. doi: 10.2196/60918 (PMC11632291; doi:10.2196/60918)
Supplement: Multimedia Appendix 2 [file formative_v8i1e60918_app2.docx]

*Severity ranking scale results from expert testers.*

| Usability heuristic | Median score^a^ | Severity rating | Score range | Evaluator recommendation for the app |
| --- | --- | --- | --- | --- |
| Visibility of system status | 0.5 | Not a usability issue | — | - “The mood tracker header doesn’t match the other headers. So, I’m not sure as a user what’s most important or if there is a hierarchy at all. It seems like the mood tracker is most important and unless I scroll all the way down, I don’t know that there are other things I could track.” - “One minor cosmetic issue identified is consistently in the headers. For example, home/activities and tools became home/tools/SMART. For me it was intuitive where I was at in the app, but it might not be for others.” |
| Does the app design clearly communicate its state? | 1 | Cosmetic issue only | 0-4 |  |
| Is app feedback presented quickly after user actions? | 0 | Not a usability issue | 0-3 |  |
| Match between system and the real world | 1 | Cosmetic issue only | — | - “Feels very familiar. See my notes about the headings on the home page. Mood tracker seems set apart from the others. This might be intentional but feels like it’s special treatment that design-wise matches the quote at the bottom more than the other cards on this page.” - “One area to consider revising to improve the natural/logical order of concepts within the app is within the ‘Main Navigation’ menu. I had a hard time teasing apart what would be things that were generally informational about the app, versus what I was supposed to do the most frequently, and what might be supportive material that would be nice to do as an additional resource. For me, it could make more sense the order of navigation as: Home, My Profile, How to Use the App. Then My Dashboard, My Activities and Tools, My Mood Tracker, Peer Chat, My Modules, My Resources, AI Support. Of note, I did see that the intent was for the app to be used in an individualized way, so may offering the ability to arrange the icons in a meaningful way to each participant might also fit with the conceptual underpinnings of the app.” |
| Will users be familiar with the terminology used in the design? | 1 | Cosmetic issue only | 0-4 |  |
| Do the design’s controls follow real-world conventions? | 1 | Cosmetic issue only | 0-3 |  |
| User control and freedom | 0 | Not a usability issue | — | - “Exit links were inconsistent—once I clicked ‘Exit’ and was taken to ESPN, other times I clicked ‘Finish,’ and it would return me to the module. For consistency/discoverability it might be helpful to have an ‘exit’ link that always takes participants to the same spot. I was unable to determine if I could cancel all actions. For example, I could edit ‘cancel’ a SMART goal entry, but I could not figure out how to change my mood documentation. I did not see that undo/redo were supported, but I also do not think this would be a significant problem for this app design.” - “Undo and redo are not supported/apparent.” - “Exit can be misconstrued as ‘log out’. I mainly used the tab on left to go back or clicking the current location (e.g., Home/Tools/Risk) to undo and redo.” - “There is an ‘Exit’ button at the bottom right, but when I click it, it goes to espn.com. I cannot go back to the app. I would think the ‘Exit’ button means to log off and it goes to the homepage. If I use the back button on my browser, it goes back, and I have to log in again (which is okay and probably preferred to protect my account). I also see breadcrumbs at the top for most of the areas, like modules, resources, activities, but not for mood tracker. During testing, I mostly used the links on the left-hand side to go back to the ‘home’ page of that section I was on but also noticed I can if I went back using the breadcrumbs pathway on the top.” |
| Does the design allow users to go back a step in the process? | 0 | Not a usability issue | 0-3 |  |
| Does the design allow users to go back a step in the process? | 0 | Not a usability issue | 0-2 |  |
| Can users easily cancel an action? | 0 | Not a usability issue | 0-3 |  |
| Is Undo and Redo supported? | 1 | Cosmetic issue only | 0-3 |  |
| Consistency and standards | 1 | Cosmetic issue only | — | - “In Activities and Tools --> my tools, the webpage refers to ‘risk’: the terms do not match. I would consider renaming ‘activities and tools’ by separating them out or further defining them. I feel they are two different things, and I do not know what it does until I click on that page. For ‘My resources,’ I would think those are resources I saved that I could refer to, but it lists all resources based on my zip code. Maybe there could be something that gives you all resources to choose from and those are the saved as favorites would be in the ‘my resources.’ ‘My Mood Tracker’ is ‘Mood analysis’ when I click on the page. I would keep it consistent. I also cannot change my mood, but that could be a test environment. Under ‘My modules,’ it does not match the homepage dashboard modules. The modules are not in the same order and the dashboard shows complete, but the ‘my modules’ do not show completed.” |
| Does the design follow industry conventions? | 1 | Cosmetic issue only | 0-4 |  |
| Are visual treatments used consistently throughout the design? | 1 | Cosmetic issue only | 0-4 |  |
| Error prevention | 1 | Cosmetic issue only | — | - “No issues with error prevention identified. The ‘hard stops’ to review (e.g., group rules that appeared the first time I entered the chat) were very helpful to review. I also received a ‘hard stop’ to review prior to deleting a goal.” - “When doing the test, such as with the safety checklist tools, it would be great if the system provided an error message when the users didn’t complete all items before saving an assessment because it might affect their overall score and interpretation.” |
| Does the design prevent slips by using helpful constraints? | 1 | Cosmetic issue only | 0-4 |  |
| Does the design warn users before they perform risky actions? | 1 | Cosmetic issue only | 0-4 |  |
| Recognition rather than recall | 1 | Cosmetic issue only | — | - “On some pages there are breadcrumbs, which are amazing. On others (mood analysis), there are not and as a I user I do not know where to go next or how to get back to where I previously was. Suggest breadcrumbs at the top of the mood analysis. Also, be consistent with your naming (it’s called mood visualization and then mood analysis).” - “To improve consistency, it would be helpful if the header labels appeared for all menu items—for example they did not appear for Mood Analysis or Peer Chat (i.e., did not show me Home/Mood analysis at the top of the page.” - “Consider pages that scroll and appear the same- e.g. AI chat is different than Peer Chat. Likewise, module on dashboard is scroll right/left, but in Modules in up/down.” - “There are help buttons on the left-hand menu, and the buttons on the menu are easy enough to get to and understand without the need to memorize.” |
| Does the app design keep important information visible, so that users do not have to memorize it? | 1 | Cosmetic issue only | 0-2 |  |
| Does the app design offer help in context? | 1 | Cosmetic issue only | 0-4 |  |
| Flexibility and efficiency of use | 0.5 | Not a usability issue | — | - “Allowing user to customize the ‘My Dashboard’ area could help to improve functionality, as well as, customizing the order of the Main Navigation.” - I do not see any shortcuts or ways to access any favorites. I cannot customize what I want to see, like on my dashboard. Maybe there could be a way to set this in my preferences or settings so there are customized options. - “In Dashboard, it would be great to add the searching feature under ‘expand your skills’ menu.” |
| Does the design provide accelerators like keyboard shortcuts and touch gestures? | 0 | Not a usability issue | 0-2 |  |
| Is app content and functionality personalized or customized for individual users? | 1 | Cosmetic issue only | 0-3 |  |
| Aesthetic and minimalist design | 1 | Cosmetic issue only | — | - “Look polished and good. However again, for PC - not up to the industry standard. Would need cosmetic updates (coherent sizing, icons locations, etc.)” - “It could be using this on my laptop rather than my phone but when I go into the modules, the text sizes are inconsistent, or the formatting is sometimes off. I do think, however, that the content is visually pleasing and designed with minimal distractions that are not relevant to the intent of the app.” - “It would be great to remove the underlying lines under each module topic.” |
| Is the app visual design and content focused on the essentials? | 1 | Cosmetic issue only | 0-3 |  |
| Have all distracting, unnecessary elements been removed? | 1 | Cosmetic issue only | 0-2 |  |
| Help users recognize, diagnose, and recover from errors | 0 | Not a usability issue | — | - “I did not see any error messages. I like the ‘are you sure’ smart goals before deleting.” - “When I enter a zip code in My Resources, no unique data are generated. Why is there not an error message or explanation? I did notice that Chicago info was retrieved with a 60007. Consider when local info is not available for a user, perhaps the next closest information could be provided. When content is not available, including in other areas of the app, why is there not a message explaining that or an error message stating something is wrong with the app? I’m left wondering if I should reload the page/app or attempt to problem solve, such as check my internet connection.” |
| Does the app design use traditional error message visuals, like bold, red text? | 0 | Not a usability issue | 0-4 |  |
| Does the app design offer a solution that solves the error immediately? | 0 | Not a usability issue | 0-4 |  |
| Help and documentation | 1.5 | Cosmetic issue only | — | - “I saw the how to use this app at the bottom of the main page and in the menu, which I think is good, but it’s really an ‘About’ page and less detailed steps about using the app itself.” - “I considered the ‘how to use the app’ to be the ‘help’ feature. It would be helpful to have ‘help’ infographics on the My Mood Tracker and My Activities and Tools tabs to provide additional information about how to use these areas within the app. I’m not sure that the ‘daily mood factors’ graphic would be immediately understandable if additional explanation about the data points and axis means.” - “I found myself scrolling around the app after a while and wondering if there is a recommended next step. Consider offering guidance to newer users at time point (e.g. 1 hour on the app) to additionally orient or guide them toward next/recommended steps (e.g. do modules).” - “I refer ‘how to use this app’ as help document - which leads to ‘About Brotherly ACT’. While it’s definitely helpful to know that the app is about - the app could benefit immensely from having a dedicated help page with corresponding interactive link that leads to what users are specifically looking for in the app.” |
| Is “help documentation” easy to find? | 1 | Cosmetic issue only | 0-2 |  |
| Is help provided in context right at the moment when the user requires it? | 2 | Minor usability issue | 0-4 |  |

^a^Heuristic scoring range: 0=none, 1=cosmetic, 2=minor, 3=major, and 4=catastrophic.
